# Hippocampal area CA2: interneuron disfunction during pathological states

**DOI:** 10.3389/fncir.2023.1181032

**Published:** 2023-04-27

**Authors:** Rebecca A. Piskorowski, Vivien Chevaleyre

**Affiliations:** ^1^Université Paris Cité, INSERM UMRS 1266, Institute of Psychiatry and Neuroscience of Paris, GHU Paris Psychiatrie et Neurosciences, Paris, France; ^2^Institute of Biology Paris Seine, Neuroscience Paris Seine, CNRS UMR 8246, INSERM U1130, Sorbonne Université, Paris, France

**Keywords:** hippocampal area CA2, inhibitory transmission, 22q11.2DS, epilepsy, multiple sclerosis, autism spectrum disorder, Alzheimer’s disease, schizophrenia

## Abstract

Hippocampal area CA2 plays a critical role in social recognition memory and has unique cellular and molecular properties that distinguish it from areas CA1 and CA3. In addition to having a particularly high density of interneurons, the inhibitory transmission in this region displays two distinct forms of long-term synaptic plasticity. Early studies on human hippocampal tissue have reported unique alteration in area CA2 with several pathologies and psychiatric disorders. In this review, we present recent studies revealing changes in inhibitory transmission and plasticity of area CA2 in mouse models of multiple sclerosis, autism spectrum disorder, Alzheimer’s disease, schizophrenia and the 22q11.2 deletion syndrome and propose how these changes could underly deficits in social cognition observed during these pathologies.

## 1. Introduction

Several decades of human and animal lesion experiments have established that the hippocampus plays a central role in learning and memory processes (Squire and Wixted, 2011). Lorente de No (1934) defined the hippocampal subregions over 80 years ago using morphology and input targets to differentiate areas CA3, CA2, and CA1. Currently, these hippocampal subregions are now delineated by many additional criteria, including molecular expression profiles (Lein et al., 2004, 2005; Cembrowski et al., 2016). With this, prominent and long-standing models have attributed particular roles to these subregions in hippocampal-dependent learning processes. For instance, pattern separation is thought to occur in the dentate gyrus, with the large numbers of low-firing dentate granule cells forming unique representations of entorhinal cortical input, and pattern completion occurring in area CA3, with the highly plastic and recurrent network allow for auto-associative properties (Willshaw and Buckingham, 1990; McClelland et al., 1995; Lisman, 1999; Rolls, 2010, 2013). This review focuses on area CA2, a region that had been under-studied, but is now recognized as playing a key role in social recognition memory (Hitti and Siegelbaum, 2014; Stevenson and Caldwell, 2014). Furthermore, as more is learned about area CA2 and how it participates in hippocampal function, it is highly likely that the neurons in this small region play a role in memory formation in non-social learning as well (Wintzer et al., 2014; Kay et al., 2016; Boehringer et al., 2017; Stöber et al., 2020; He et al., 2021; Lehr et al., 2021). Even though neurophysiologists have neglected hippocampal area CA2, neuroanatomists have consistently noted changes in this region in post-mortem tissue from patients with neurodegenerative or psychiatric disorders. These results have been assembled and previously reviewed (Chevaleyre and Piskorowski, 2016). Here, we aim to present recent results from mouse models demonstrating how detrimental changes in inhibitory transmission and plasticity in area CA2 underly the cognitive deficits displayed in these models, and potentially lead to better understanding of hippocampal dysfunction in patients.

## 2. Unique inhibitory transmission and synaptic plasticity in hippocampal area CA2

Interneurons play a critical role in tuning the balance between excitation and inhibition in all brain areas. If it can be said that one area relies more heavily on inhibitory transmission than any other, then area CA2 may qualify as a winner of the contest. In comparison to areas CA1 and CA3, there are very few studies examining the cellular composition of hippocampal area CA2. The pyramidal neurons in this region have larger soma than CA1 and dendritic arborizations that more closely resemble CA3 pyramidal neurons, except that CA2 pyramidal neurons lack the distinctive thorny excrescences that form the post-synaptic contact with mossy fibers from dentate granule cells (Ishizuka et al., 1995). There is some functional evidence that deep and superficial CA2 pyramidal cells have different firing properties during exploration (Kay et al., 2016; Oliva et al., 2016a,b). However, the cellular properties and circuitry allowing this difference to emerge are unknown.

Several anatomical studies have shown that area CA2 has the highest density of several classes of interneurons in the hippocampus in both rodents and primates. The remarkable density of parvalbumin-positive (PV+), and calbindin-positive cells in primate hippocampal CA2 was first reported in 1991 (Leranth and Ribak, 1991). A thorough and elegant study used alpha-actinin-2 and PCP4 to define the boundaries of CA2 in rat hippocampal sections, and carefully quantified the density of interneurons labeled with a large panel of markers in all the strata of all CA regions (Botcher et al., 2014). This study demonstrated that the density of both PV+ and Reelin-positive neurons were highest in *Stratum oriens* (so) of CA2, and that PV+, Reelin+ and Calbindin+ cells were the highest density in *Stratum pyramidale* (sp), as compared to areas CA3 and CA1. The high density of PV+ cells bodies in sp and so was also observed in mice using RGS14 staining as a boundary to define CA2 (Piskorowski and Chevaleyre, 2013). The morphology and physiological properties of GABAergic cells in area CA2 is vastly understudied as compared to CA1, where there are at least 21 different types of interneurons (Klausberger and Somogyi, 2008). Based on morphology, two distinct populations of basket cells have been identified in area CA2 (Mercer et al., 2007). Of these two classes, one closely resembles the basket cells of CA1, staining positive for either PV or CCK, with narrow spiny dendrites and axons that are restricted to nearby sp. The other class of basket cells were strikingly different, with long horizontal dendrites and extensive axonal arbors that extended far into CA1, CA2, and CA3 (Mercer et al., 2007). The bistratified cells in CA2 also displayed important differences from the bistratified cells in CA1. CA2 bistratified cells are PV+ and have dendrites that extend through so and sr without entering slm, entering into neighboring CA1 and CA3. The axons show a remarkable subfield preference, extensively innervating CA2 and CA1, but stopping abruptly at the CA3 border (Mercer et al., 2007). There is also a unique interneuron subtype that has been identified in area CA2, called SP-SR interneurons (Mercer et al., 2012). Similar to bistratified cells, these cells have soma in sp, and extend dendrites into so and sr. The axons of these cells, however, form dense arborizations that are restricted to sr (Mercer et al., 2012). These cells are not immunopositive for PV or CCK, and have intrinsic properties that are similar to basket cells. Thus, compared to hippocampal area CA1, the GABAergic interneurons are not well studied. However, what has been discovered reveals that this region has a distinctive local inhibitory network that can readily be recruited by intra-hippocampal inputs.

**FIGURE 1:**
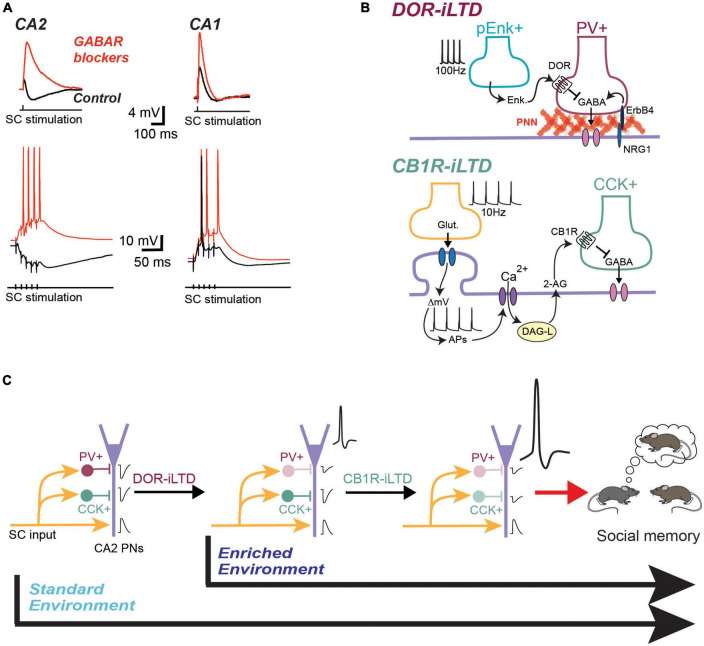
CA2 pyramidal cells receive a massive feed-forward inhibition that is plastic and modulated by environmental enrichment. **(A)** Exemple traces of synaptic responses recorded in CA2 and CA1 PN in response to CA3 input stimulation. In control condition (black traces) the inhibitory component almost completely shunts the EPSP in CA2 PNs. The inhibitory component is even more obvious during a train of stimulation of CA3 inputs. when inhibitory transmission is blocked (red traces), the EPSP becomes much larger and CA2 PN can easily fire action potentials during a train of stimulation. **(B)** Model for the DOR-iLTD and CB1R-iLTD expressed in area CA2. Top: DOR-plasticity is expressed by PV+ IN. It requires the release of enkephalins and activation of DOR located on PV+ terminals. It is also dependent on positive control of GABA release by the interaction between ErbB4 and Neureguline 1 (NRG1). This interaction is itself dependent on the PNN located around the soma of PNs. Bottom: CB1R-iLTD is expressed by CCK+ IN following activation of CB1R by the eCB 2-AG. 2-AG is released by calcium increase in the PN following stimulation of CA3 inputs and action potential firing by the PN. **(C)** Two-step model for the involvement of inhibitory plasticity in social memory formation. In mice raised in a standard environment, DOR-iLTD is induced following a single social interaction with a novel mouse. The resulting decrease in inhibition allows PNs to fire action potentials. This in turn allows the induction of CB1R-iLTD, a plasticity that is induced after several interactions with the novel mouse. Blocking CB1R in CA2 completely prevents social memory formation. In an enriched environment, DOR-iLTD is occluded and thus already induced during the enrichment. In this environmental condition, it is likely that social memory formation only requires CB1R-iLTD.

One of the remarkable aspects of the local CA2 network is the large magnitude of feed-forward inhibition that is recruited by CA3 input stimulation, especially compared to the amount of inhibitory transmission recruited in area CA1 (Figure 1). We postulate that the large density of interneurons in area CA2 allows for this large amount of feedforward inhibition that nearly entirely shunts the excitatory transmission when the cells are held at physiological resting membrane potentials. When GABA-ergic transmission is blocked, the excitatory transmission at the CA3-CA2 synapse is comparable to the CA3-CA1 synapse (Chevaleyre and Siegelbaum, 2010). Thus, when CA2 and CA1 are examined with the same method in acute hippocampal slice, there is an intriguing and important difference in the E/I ratio in these two regions with CA3 excitation. Furthermore, high-frequency stimulation of CA3 input readily results in action-potential firing in CA1 with inhibition intact. However, quite the opposite is true in area CA2, with high frequency trains resulting in a net hyperpolarization of the membrane potential.

Another puzzling property of CA2 pyramidal neurons that is independent of the local inhibitory network is the resistance of these cells to activity-induced long-term potentiation (LTP) at the excitatory CA3-CA2 synapse. This property was first uncovered and demonstrated in-depth by Zhao et al. (2007). Furthermore, it has since been established that this is due to the unique of expression of the regulator of g-protein signaling 14 (RGS14) (Lee et al., 2010; Evans et al., 2018) and calcium extrusion and buffering (Simons et al., 2009). This discovery and mechanism were quite puzzling given the important role of NMDA-mediated LTP in learning and memory in the hippocampus (McHugh et al., 1996, 2007; Poo et al., 2016).

So what gives? How can an entire hippocampal region be devoid of synaptic plasticity? While the glutamatergic synapses between CA3 and CA2 are resistant to activity-dependent plasticity, it is now clear that the inhibitory synapses in area CA2 express activity-induced synaptic plasticity that can powerfully shift the balance of excitation and inhibition and allow CA2 pyramidal neuron action potential firing. Specifically, there are two separate mechanisms allowing activity dependent long-term depression of GABAergic transmission (iLTD) in area CA2. One is mediated by the activation of delta-opioid receptors (DORs), and the other by cannabinoid type I receptors (CB1Rs). Importantly, there is evidence that both of these plasticities play a role in social recognition memory. How is this possible? Hippocampal CA2 pyramidal neurons project strongly to ventral hippocampal CA1 (Meira et al., 2018), a region that has been demonstrated by several studies to be important for social memory encoding (Okuyama et al., 2016; Rao et al., 2019; Tsai et al., 2022). By tightly regulating CA2 pyramidal neuron output, synaptic plasticity of inhibitory transmission in area CA2 results in an enhanced excitatory drive onto CA1 with CA3 activity (Nasrallah et al., 2015, 2019). CA1 pyramidal neurons provide an important output to the hippocampus, projecting to cortical regions that are also very important for social discrimination. Furthermore, CA2 pyramidal neurons also project to extra-hippocampal structures, including hypothalamic nuclei and the septum (Cui et al., 2013; Hitti and Siegelbaum, 2014; Leroy et al., 2018). These structures, are playing central roles in social cognition and complex behaviors (Walsh et al., 2021; Wu et al., 2021; Griguoli and Pimpinella, 2022).

### 2.1. Parvalbumin-expressing interneurons and DOR-mediated iLTD

The DOR-mediated iLTD that occurs in hippocampal area CA2 is specific to a subset of PV+ inhibitory neurons (Piskorowski and Chevaleyre, 2013). This was determined by selective expression of channelrhodopsin in PV+ interneuron and using light to evoke an inhibitory synaptic current while recording from CA2 pyramidal cells in voltage clamp mode. Following the application of a selective DOR agonist, the light-evoked PV-specific transmission underwent an iLTD. The same experiments performed in area CA1 resulted in a transient depression of inhibitory transmission, indicating that the DOR-mediated iLTD seen in area CA2 does not occur in CA1. It was reported later, however, that CA3 pyramidal neurons express a DOR-iLTD that is similar to area CA2 (Domínguez et al., 2019). Further evidence that the DOR-iLTD is exclusive to PV+ interneurons came from experiments in which GiDREADDs were expressed exclusively in PV+ cells. Inhibition of PV+ cells by CNO application entirely prevented induction of DOR-iLTD (Nasrallah et al., 2019). The DOR-iLTD is entirely pre-synaptic, with activation of the receptors resulting in reduced GABA release (Piskorowski and Chevaleyre, 2013). There is evidence that the resulting decrease in GABA release probability also depends on a trans-synaptic signaling between pyramidal neurons and PV+ interneurons through activation of the ErbB4 receptor located on PV+ terminals by Neuregulin 1 (Domínguez et al., 2019). This signaling is dependent on the integrity of the highly unusual and specialized perineuronal net (PNN). In many brain regions, PV+ interneurons are encased in PNNs, which are thought to regulate synaptic formation (Fawcett et al., 2019) and reduce oxidative stress (Cabungcal et al., 2013). However, in area CA2, the role of the PNN may be more complex, as this specialized extracellular matrix surrounds both PV+ interneurons as well as CA2 pyramidal cells. Furthermore, in contrast to inhibitory synapses, it has been shown the presence of the PNN in area CA2 also acts to prevent plasticity at glutamatergic synapses on CA2 pyramidal neurons (Carstens et al., 2016).

Evidence has been presented that enkephalin, the endogenous ligand for DORs, is potentially released by local vasoactive intestinal peptide-expressing (VIP+) interneurons (Leroy et al., 2017). DOR-iLTD can be induced by high-frequency, theta burst and 10 Hz stimulation of CA3 inputs (Piskorowski and Chevaleyre, 2013). Furthermore, a DOR-mediated iLTD in area CA2 has also been proposed to be inducible by a narrowly timed pairing of CA3 and distal cortical input activity, called input timing dependent plasticity (ITDP) (Leroy et al., 2017). Thus, the VIP+ cells releasing enkephalin are likely receiving input from both CA3 and cortical terminals.

There is evidence that the DOR-mediated iLTD in area CA2 contributes to social memory formation but may not be absolutely required. Selective removal of DORs in area CA2 does impair social recognition memory as tested by a 5-trial dishabituation task (Domínguez et al., 2019). In this experiment, the mice explored a familiar mouse more on the second and third exposure, but less on the fourth, indicating that with enough trials, social memory formation could occur. Similar results were observed with localized infusion of DOR-receptor antagonist into areas CA2 and CA3 (Leroy et al., 2017). Likewise with localized digestion of the PNN in area CA2, which prevents DOR-iLTD, social recognition memory was impaired, but not completely abolished (Domínguez et al., 2019). Interestingly, it was observed that the PV+ inhibitory network undergoes a maturation in area CA2 in late adolescence/early adulthood, with both the DOR-iLTD and basal ErbB4 activation emerging at ∼5 weeks after birth, around when mice start to display preference for novel conspecifics (Domínguez et al., 2019). Interestingly, though, it has been shown that very young mice need functional hippocampal CA2 activity in order to differentiate their mother from other female conspecifics (Laham et al., 2021). Thus, from these data, it seems that GABAergic plasticity at PV+ neurons in area CA2 may contribute to social memory formation but is clearly not the only mechanisms at play.

The PV+ interneurons in hippocampal area CA2 receive very strong excitatory input from the hypothalamic supramammillary nucleus (SuM) (Robert et al., 2021). This hypothalamic nucleus targets specifically the dentate gyrus and hippocampal area CA2 (Haglund et al., 1984). Strongly activated by novelty (Wirtshafter et al., 1998; Ito et al., 2009) and locomotion (Farrell et al., 2021) the SuM sends two separate projections to the dentate gyrus and hippocampal area CA2 (Chen et al., 2020). The SuM cells that project to area CA2 show increased c-fos and action potential firing with social novelty exposure. Furthermore, optogenetic activation of the CA2-projecting SuM cells increases the exploration of familiar conspecifics, supporting the hypothesis that this input conveys a novelty signal (Chen et al., 2020). Use of optogenetics and acute hippocampal slices revealed that the SuM input stimulation drives a very strong feed-forward inhibition onto CA2 pyramidal cells that not only controlled the intrinsic firing properties of CA2 pyramidal cells, but also had a significant impact on area CA1 activity via CA2 (Robert et al., 2021). Many experiments in this work provide evidence that PV+ interneurons are the primary target of the SuM hypothalamic inputs, and that the same cells that undergo the DOR-iLTD are also receiving SuM input. Thus, the PV+ neurons in area CA2 may not only be regulating the input from CA3 but are also clearly playing an important role in gating extra-hippocampal hypothalamic inputs.

### 2.2. Endocannabinoid-mediated depression of inhibitory transmission in area CA2, CCK+ interneurons

It has recently been reported that endocannabinoid-mediated synaptic plasticity is playing an important role in hippocampal area CA2 function. This plasticity results from the release of endocannabinoids (eCBs) by CA2 pyramidal cells and activation of cannabinoid type 1 receptors (CB1Rs) located on GABA-ergic terminals (Loisy et al., 2022). As established in CA1, the CB1Rs are likely expressed by CCK+ interneurons in area CA2. This is supported by the observation that blocking N-type calcium channels involved in GABA release from CCK+ terminals prevented this plasticity and that the chemogenetic silencing of PV+ interneurons did not alter CB1R-mediated plasticity (Loisy et al., 2022). The mechanism underlying the plasticity CB1R-iLTD in area CA2 is very different from what has been found in area CA1 (Figure 1B; Chevaleyre and Castillo, 2003). In CA1 pyramidal cells, the release of eCB is induced by the activation of type-I metabotropic glutamatergic receptors, activation of phospholipase C, leading to diacylglycerol (DAG) and activation of DAG-lipase activation, which results in release of the endocannabinoid 2-Arachidonoylglycerol (2-AG). This plasticity is extremely localized to the dendritic region surrounding the CA3 terminals responsible for the post-synaptic activation of mGluR-I, making this iLTD very input-specific. However, it will also likely occur on neighboring inhibitory synapses of CA1 pyramidal cells sharing the same CA3 input (Chevaleyre et al., 2006). In CA2 pyramidal cells, action potential firing of the pyramidal cell alone is sufficient to allow sufficient calcium entry though voltage-activated calcium channels to lead to the activation of DAG-lipase and release of 2-AG along the entire dendritic arbor. Thus, this eCB-mediated iLTD is not input specific, as all GABAergic inputs with CB1Rs will undergo iLTD. However, it is very cell-specific, allowing only the CA2 pyramidal cells that were sufficiently recruited by synaptic input to fire action potentials to undergo further amplification, thus rising above the noise and having a stronger influence on the hippocampal network (Loisy et al., 2022). Thus, normally, as shown in Figure 1, CA3 input stimulation results in a large hyperpolarization of CA2 pyramidal cells because of recruited feed-forward inhibition from the PV+ network, and the eCB plasticity does not occur. However, following prior induction of DOR-iLTD, CA2 pyramidal cells can fire action potentials with CA3 stimulation (Nasrallah et al., 2015, 2019). The requirement of AP firing by CA2 pyramidal cells leads to a two-steps model where DOR-iLTD of the PV+ network is permissive for the induction of CB1R-iLTD (Figure 1C). Critically, this model also suggests that CB1R-plasticity may be enabled after any physiological change that enables AP firing in CA2 pyramidal cells, whether is it through a decrease in inhibitory transmission (i.e., iLTD), an increase in excitatory transmission (i.e., LTP) or a change in pyramidal cell intrinsic excitability by neuromodulators such as oxytocin (Tirko et al., 2018).

We hypothesize that the eCB-iLTD is critical and necessary for area CA2 to allow for social memory formation, and likely playing a more prominent role than the DOR-iLTD. The DOR-mediated iLTD was found to be occluded after a single social interaction (Leroy et al., 2017; Loisy et al., 2022). However, as mentioned earlier, blocking DOR-iLTD by several strategies only impedes social memory formation, but does not prevent it. In contrast, CB1R-iLTD is occluded after four social interactions (Loisy et al., 2022). Furthermore, social memory formation was shown to be completely impaired even after 4 presentations of a familiar conspecific when CB1 R antagonist was locally infused into area CA2 (Loisy et al., 2022). Additional evidence for the central importance of the CB1R-iLTD in social memory comes from the recent finding that DOR-plasticity cannot be induced after mice have spent 3 weeks in an enriched environment (EE) (Loisy et al., 2023). In fact, we postulate that the DOR-iLTD is occluded after EE. Evidence for this come from the reduced GABA release and neither DOR activation or ErbB4 block (two pathways that modulate GABA release from PV+ INs) have significant effect on GABA transmission in EE-housed mice. It is well-established that 3 weeks in an EE environment enhances many aspects of hippocampal-dependent learning, improving performance in numerous animal models of neurodegenerative and psychiatric disorders (Hannan, 2014). Interestingly, DOR-plasticity can be induced in EE-housed animals following 24 h of social isolation (Loisy et al., 2023). Thus, it is possible that he PV+ inhibitory network in area CA2 may be linked to internal state. When mice are stressed or raised in an impoverished environment, transmission from PV+ INs increases, thus allowing DOR-iLTD to be induced and preventing the recruitment of CA2 pyramidal cells by CA3 activity. CA2 pyramidal cells do not fire action potentials and social recognition memory is impaired. This is in fact what is seen when social recognition memory is tested in socially isolated animals (Kogan et al., 2000). Thus, as numerous psychiatric disorders and neuropathologies are linked to stress-induced worsening of symptoms, it seems fitting that the inhibitory network in area CA2 will be compromised.

## 3. Alterations of inhibitory transmission in area CA2 during pathological states

Given the critical role of inhibitory transmission and plasticity in controlling pyramidal cell firing in area CA2, it is not surprising that inhibitory transmission has been found to be altered in several neurodevelopmental and neurodegenerative diseases (Chevaleyre and Piskorowski, 2016). While most early findings on area CA2 where derived from studies on human tissue (Williamson and Spencer, 1994; Benes et al., 1998), recent evidence using mouse models of diverse pathologies have provided a more detailed descriptions of the alterations present in area CA2. In the following sections, we summarize the changes that are observed in area CA2 during several pathologies where the function of inhibitory networks is clearly impaired.

### 3.1. Multiple sclerosis

**FIGURE 2:**
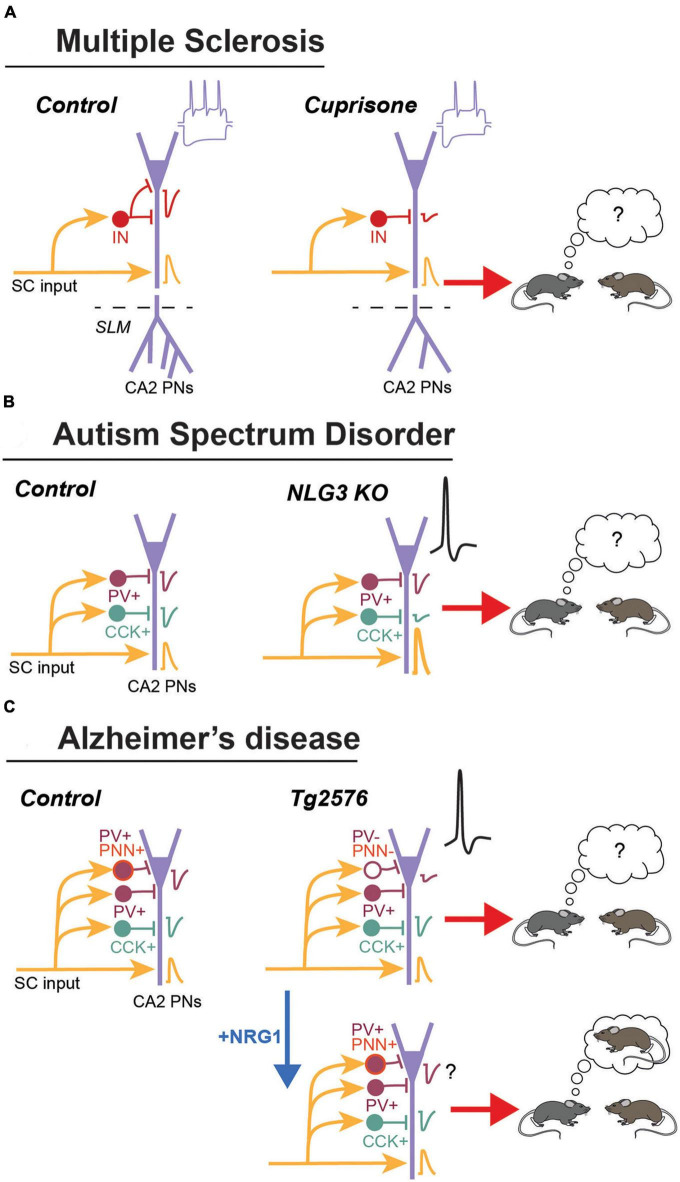
Summary of the main changes observed in area CA2 during MS, ASD and AD. **(A)** In the cortisone mouse model of demyelination. These mice display an accumulation of the C1q component of the complement around inhibitory synapses in CA2. Excitatory transmission from CA3 (Shaffer collaterals: SC) is normal but inhibitory transmission is reduced. The distal dendritic branching is also reduced. Pyramidal neurons have and increased sag during hyperpolarization and fire less action potential. This lower PN excitability could explain the deficit in social memory formation. **(B)** In the Neuroligin 3 KO mice, excitatory transmission for CA3 is increased. Inhibitory transmission from CCK+ but not from PV+ IN is reduced. This resulted in an increased excitability and bursting activity of PNs *in vivo*. This mice have a deficit in social memory formation. **(C)** In the Tg2576 mouse model schizophrenia, there is a decrease in PV and in the PNN expression. This could explain the decrease in inhibitory transmission and DOR-plasticity, as well as the large increase in action potential firing by PNs. This mice display a strong deficit in social memory formation. Injection of Neuregulin 1 in area CA2 restores PV and PNN expression, as well as social memory formation.

Multiple sclerosis (MS) is an autoimmune disease often associated with severe cognitive symptoms including deficits in social cognition and facial emotion recognition. Interestingly, these symptoms can be observed early in the disease progression, even before appearance of other cognitive symptoms (Pöttgen et al., 2013). Some of the deficits have been linked to a structural and functional disconnection of the hippocampus from several brain networks, a change that might result from an aberrant loss of synapse during synaptic pruning and extensive demyelination. Components of the complement such as C1q and C3, which are part of the immune system, have been implicated in the process of pruning during development but also in re-shaping neuronal networks during forgetting in adults (Schafer et al., 2013; Wang et al., 2020). In the hippocampus of post-mortem MS patients, the C1q and C3 components accumulate predominantly in the CA2/3 area at synapses that are engulfed by microglial processes (Michailidou et al., 2015). C1q accumulation in CA2 was also shown to be higher in patients with cognitive impairment compared to those with normal cognition (Ramaglia et al., 2021). Using the cuprizone model of demyelination, this study also characterized the changes occurring in area CA2 (Figure 2A). C1q accumulation was larger in the CA2 pyramidal layer and was colocalized with inhibitory synapses engulfed by microglia/macrophages. Stimulation of CA3 input led to normal excitatory transmission but feed-forward inhibitory transmission was strongly reduced. In agreement with a loss of inhibitory synapses, a decrease in the number of puncta for the GABA transporter VGAT was also observed in the pyramidal layer. However, pyramidal cells fire less action potentials in response to trains of stimulation of CA3 inputs (in presence of blockers of GABA transmission) and to depolarizing steps. Pyramidal cells also display a larger sag during hyperpolarizing steps, indicating an increase in the current mediated by HCN channels. Finally, in agreement with the impaired inhibition and CA2 pyramidal cell excitability, this study reported that social recognition memory was strongly impaired in this mouse model of MS. Altogether, this study reveal how accumulation of the C1q component of the complement in area CA2 can result in memory deficits in mice but also potentially in humans.

### 3.2. Autism spectrum disorders

Autism spectrum disorders (ASD) include neurodevelopmental diseases characterized by deficits in verbal and non-verbal communication, stereotyped behaviors and impaired social cognition. Many genes involved in synaptic function have been linked to the development of ASD, including mutations or deletions in genes encoding for Neuroligins (NGLs). NGLs are expressed by the postsynaptic elements and interact with their presynaptic partners Neurexins. Mice lacking NGL3 have been used as a model for a non-syndromic form of autism and NGL3 KO mice display strong deficits in social behaviors.

Using electrophysiological recordings *in vivo* and *ex vivo*, a recent study performed with these mice has focused on the potential alterations in hippocampal area CA2 (Modi et al., 2019). First, using extracellular recordings *in vivo*, this study reported a significant reduction in oscillatory activity in the theta and gamma frequencies in both areas CA3 and CA2, as well as a more specific decrease in the amplitude of local field potential oscillation recorded in CA2. Using juxtacellular recordings of putative pyramidal cells, a large increase in spike frequency as well as bursting activity was reported in area CA2 but not in CA3. This data suggested an increased excitability specific to CA2 pyramidal cells (Figure 2B).

To address more precisely the change in CA2 activity, the authors used whole cell recordings in hippocampal slices. These experiments revealed that the frequency but not the amplitude of isolated spontaneous EPSCs (recorded in presence of blockers of GABA transmission) was increased in CA2 pyramidal cells (Modi et al., 2019). Conversely, isolated spontaneous IPSCs frequency (but not amplitude) recorded in CA2 pyramidal cells was decreased in NLG3 KO mice. To address which class of interneuron might be responsible for the decrease in inhibitory drive, the authors used the fact that GABA release from PV+ and CCK+ interneurons is mediated by different calcium channels (respectively, P/Q and N types). They found that the component of evoked IPSCs abolished by a blocker of N type channels was strongly reduced in NLG3 KO mice, while the component of IPSCs dependent on P/Q type channel was unaltered. These data indicated that inhibitory transmission from CCK+ INs, but not from PV+ INs, was reduced in the NLG3 KO mice. However, quantification of PV+ and CCK+ puncta in the pyramidal layer of CA2 suggested that the number of synapses from these 2 classes of interneurons was not altered, suggesting that the reduced inhibitory transmission resulted from a decrease in GABA release probability and not from a reduced number of synapses.

Finally, this study also shows that NLGN3 KO have a deficit in social memory formation, i.e., they spend the same amount of time exploring a novel and a familiar mouse. However, while these mice might indeed have an impairment in the capacity to remember a conspecific, an effect that would be consistent with a disfunction in the CA2 network, caution should be taken when interpreting this result. Indeed, the same study clearly showed that NLGN3 KO mice also have a deficit in sociability, an effect that could potentially result from a deficit in olfactory functions as described previously in a NLGN3-deficient mice (Radyushkin et al., 2009). Thus, it is unclear whether the apparent lack of social memory results from an impairment in remembering a familiar conspecific, or just from a general alteration in sociability. In any case, this study convincingly shows that alteration in CA2 inhibitory transmission from a specific class of interneuron can result in more global disfunction of the CA2 network, thus likely contributing to some of the hippocampal-dependent phenotypes observed in this mouse model of ASD.

### 3.3. Alzheimer’s disease

Alzheimer’s disease (AD) is a neurodegenerative disease leading to dementia in the elderly population. Symptoms include impairments in acquisition and retrieval of memories that are linked to alterations in hippocampal function (Hirjak et al., 2017). In particular, the inability to remember and recognize other individuals, including family members, is a particularly strong burden on patients. This deficit in social recognition memory suggested that area CA2 might be altered during AD (Figure 2C). Indeed, the largest and earliest decrease in INs expressing PV and surrounded by the PNN in the hippocampus is observed in area CA2 of the Tg2576 mouse model of AD (Cattaud et al., 2018).

A recent study focused on area CA2 has shown that PV+ and PNN+ cell density is strongly reduced in area CA2. This was accompanied by a large decrease in inhibitory transmission between CA3 and CA2, leading to a large increase in EPSP amplitude and the emergence of action potential firing in CA2 PNs in response to CA3 input stimulation. Impaired inhibitory transmission from PV+ interneurons likely contribute to the reduced inhibitory transmission because DOR-iLTD as well as the DOR-mediated disinhibitory increase in PSP amplitude was strongly reduced in this mice. In agreement with a role of this plasticity in social memory formation, social recognition memory was completely abolished in Tg2576 mice.

Interestingly, degradation of the PNN following ChABC injection in area CA2 also completely prevented social memory formation (Domínguez et al., 2019; Rey et al., 2022). The PNN in area CA2 was previously shown to be required not only for social memory formation, but also for the induction of DOR-iLTD onto PV+ IN, likely by allowing a *trans*-synaptic signaling through interaction between the Neuregulin 1 (expressed by PNs) and its receptor ErbB4 expressed by PV+ INs (Figure 1). degradation of the PNN prevents the positive control of ErbB4 on GABA release probability, a mechanism that could potentially underly the decreased inhibitory transmission in Tg2576 mice. Accordingly, restoring activation of the ErbB4 receptor via a local injection of NRG1 in area CA2 was found to also restore some of the deficits observed in Tg2576 mice. Not only PV and PNN expression were found to be normalized after NRG1 injection, indicating that PV+ INs were not dead but just expressing less PV, but also social memory formation was completely rescued. While inhibitory transmission and plasticity was not tested after NRG1 injection, one can speculate that increasing ErbB4 receptor activation in PV+ INs will also restore GABA release probability and induction of DOR-iLTD from PV+ cells, hence re-allowing the emergence of sequential inhibitory plasticities and the formation of social recognition memory.

Independently of the exact mechanism engaged following NRG1 injection, it is striking to consider that in a pathology affecting many brain structures, a manipulation of excitability specifically in area CA2 was sufficient to fully restore the formation of social memory in Tg2576 mice. This further strengthen the critical role of this area and of inhibitory transmission and plasticity in the formation of social memory in the normal brain, but also the contribution of this area in the development of cognitive symptoms in pathologies leading to impairments in social cognition. It is also interesting to note that a transient exposure of Tg2576 mice to and enriched environment had lasting beneficial effect on PV/PNN expression and memory formation. Although social memory was not tested after enrichment in Tg2576 mice, one can speculate that it could also be improved, once again though a normalization of PV+ IN synaptic transmission and DOR-mediated plasticity (Cattaud et al., 2018).

### 3.4. Epilepsy

One of the earliest study on area CA2 revealed that this region might play an important role in the pathology of epilepsy (Williamson and Spencer, 1994). Performed on hippocampal samples from epileptic patients, this study revealed that the vast majority of CA2 PNs recorded in current clamp display no apparent inhibitory transmission. The same study also observed an excitatory transmission between granule cell mossy fiber’s axons and CA2 PNs in epileptic tissues from medial temporal lobe sclerosis patients but not from tumor-associated temporal lobe epilepsy patients. Other studies also performed in human tissue from epileptic patients confirmed the important decrease in spontaneous inhibitory transmission (Wittner et al., 2009) and the sprouting of mossy fiber terminals (Arellano et al., 2004; Freiman et al., 2021) with the appearance of excitatory synapses on the soma of CA2 PNs resembling mossy fiber boutons (Wittner et al., 2009). Several studies from human tissues also observed that the density of PV+ IN was reduced in area CA2 of epileptic samples (Arellano et al., 2004; Andrioli et al., 2007; Wittner et al., 2009), and this decrease was ascribed potentially to a reduced expression of PV by INs rather than their death (Wittner et al., 2009).

Subsequent studies performed on mouse models of Temporal Lobe Epilepsy (TLE) have confirmed key data observed in human tissues from epileptic patients and also provided a detailed description of the anatomical and functional changes observed in area CA2 during the course of the disease. Specifically, aberrant mossy fiber sprouting in area CA2 was observed in the kainate mouse model of TLE (Häussler et al., 2015; Freiman et al., 2021). This extra connectivity is likely functional as *in vivo* recording revealed epileptic activities in CA2 that were shortly preceded by epileptic activities in the dentate gyrus (Häussler et al., 2015). Subsequent studies using unilateral kainate injection has shown that CA2 plays an active role in the generation of epileptic activity and in the propagation of these activity to the non-sclerotic contralateral hippocampus, including the contralateral CA2 (Kilias et al., 2022). Another study performed on the Pilocarpine mouse model of epilepsy confirmed that DG-CA2 PN excitatory transmission was increased and that feed-forward inhibitory transmission from CA3 and from CA2 recurrent connections were decreased (Whitebirch et al., 2022). Furthermore, reducing the hyper-excitability of CA2 PNs following inhibitory DREADD expression in Amigo2-cre+ mice was sufficient to reduce the number but not the duration of convulsive seizures in pilocarpine treated mice (Whitebirch et al., 2022).

**FIGURE 3:**
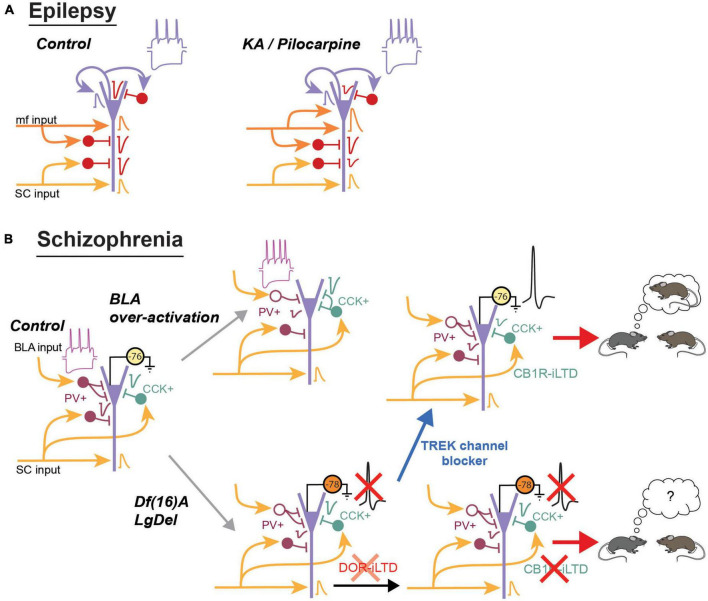
Summary of the main changes observed in area CA2 during schizophrenia and epilepsy. **(A)** In the kainate model of epilepsy, there is a sprouting of the mossy fibers terminals in area CA2 and *in vivo* recordings indicate an increased connectivity between the dentate gyrus and CA2. In the pilocarpine mouse model, excitatory transmission from the dentate gyrus is increased. Conversely, feed-forward inhibitory transmission from CA3 and from CA2 is reduced. PNs also have an increased sag during hyperpolarization and fire more easily action potentials. **(B)** The mouse model of schizophrenia resulting from over-activation of the BLA display a decrease in PV expression and inhibitory transmission. The number of PV+ terminals is also reduced while the number of CCK+ terminals is increased. IN in SO display an increase in their sag and an increase in action potential firing frequency. In the Df(16)A or LgDel mouse model of schizophrenia, the number of PV+ IN is reduced and inhibitory transmission is decreased. DOR-iLTD is reduced and because PNs are also more hyperpolarized, they do not fire action potentials following DOR-plasticity. As a consequence, CB1R-iLTD is also strongly reduced. Blocking TREK channels depolarizes PNs and allow them to fire action potentials. This depolarization is sufficient to restore CB1R-iLTD and social memory formation.

Epilepsy has long been thought to result from an imbalance between excitation and inhibition. Although anatomical and functional changes have been observed in all hippocampal areas, CA2 is unique in that it is much more resistant to cell death in comparison to area CA1 or CA3. Furthermore, while acutely reducing inhibitory transmission in rodent hippocampal slices was by itself sufficient to trigger the generation of epileptic-like activities that originated in area CA2 (Wong and Traub, 1983; Knowles et al., 1987), it is likely that both the increased excitatory drive from mossy fiber sprouting and the reduced inhibitory transmission cooperate in the generation of spontaneous interictal-like activity observed in area CA2 of TLE patient (Wittner et al., 2009). Therefore, both an increase in excitation and a decrease in inhibition in this region contribute to strongly shift the E/I balance toward a hyper-excitable and pathological state (Figure 3A).

### 3.5. Schizophrenia and the 22q11.2 deletion syndrome

Early studies on post-mortem hippocampal tissues from schizophrenic patients have reveal that CA2 is uniquely altered. In particular, a large decrease in the density of interneurons was reported in area CA2 during schizophrenia (Benes et al., 1998; Zhang and Reynolds, 2002). A meta-analysis reported that a decrease in PV+ INs in area CA2 was one of the very few parameters significantly changed in the hippocampus during schizophrenia (Knable et al., 2004).

A decrease in interneuron density in area CA2 was also reported in several rodent models of schizophrenia. One model consists in transiently over-activating the basolateral amygdala (BLA) inputs that project to the basal dendrites of CA2 and CA3 pyramidal cells. This manipulation during adolescence results in a lasting decrease in the density of interneurons expressing PV, calretinin and calbindin located in *Stratum oriens* of CA2 and CA3 (Gisabella et al., 2009). The density of basket terminals arising from PV+ interneurons was also reduced. This anatomical changes were accompanied by a decrease in both spontaneous inhibitory currents and IPSCs evoked by stimulation in *Stratum radiatum* (Gisabella et al., 2005). Furthermore, these studies also show that the intrinsic properties of remaining interneurons in so were altered. In particular, an increase in 1h (current that mediates the sag observed during hyperpolarization of the cell), a decrease in action potential duration and an increase action potential firing frequency were observed following over-activation of the BLA (Gisabella et al., 2009).

Changes in interneuron properties have also been observed in mouse models of the 22q11.2 deletion syndrome (Figure 3B). This spontaneous deletion of around 30 genes is one of the highest genetic factors for schizophrenia diagnosis (Drew et al., 2011). Mice that have an equivalent genetic deletion on chromosome 16 have been generated [Df(16)A and LgDel mice]. Similar to the social cognition deficits observed in humans, Df(16)A mice have impaired social memory formation. Furthermore, they display a decreased density of PV+ interneurons in area CA2 but not in CA1 or CA3 (Piskorowski et al., 2016). This decrease in PV density was accompanied by a decrease in feed-forward inhibitory transmission from CA3 inputs, while excitatory CA3 transmission was not altered. However, despite the decrease in inhibitory transmission, CA2 pyramidal cells were found to be less likely to fire action potentials when stimulated by CA3. This is because CA2 pyramidal cells are more hyperpolarized due to an increased conductance of the TREK potassium channel that is highly expressed in area CA2 and known to control resting membrane potential (Talley et al., 2001; Honoré, 2007; Piskorowski et al., 2016). Interestingly, the decrease in PV expression, the change in membrane potential and the decrease in inhibitory transmission are age dependent: they were not altered during early adolescence (up to post-natal week 5) but started to change after this period. PV+ interneurons likely contribute to the global decrease in inhibitory transmission because DOR-plasticity is reduced in these mice. In the LgDel mice that carry a similar deletion as the Df(16)A mice, CB1R-plasticity was also shown to be impaired (Loisy et al., 2022). This impairment likely results from the reduced DOR plasticity, preventing CA3 activity from inducing DOR-iLTD in area CA2 as efficiently as in WT, and further, the resting membrane potential of pyramidal cells start from a more hyperpolarized potential, preventing action potential firing. Interestingly, if sufficient current was injected into the cells to evoke action potential firing, CA2 pyramidal cells from LgDel mice were capable of undergoing CB1R-iLTD.

*In vivo* recordings have shown that CA2 PNs can encode social information (i.e., distinction between familiar and novel mouse). In Df(16)A mice, this coding was impaired while spatial coding, initially weak in WT mice, was enhanced (Donegan et al., 2020). Furthermore this study also showed that increasing CA2 PN excitability by blocking TREK channels, either through unspecific pharmacological manipulation or through expression of a dominant negative of TREK channels specifically in CA2 pyramidal cells, was sufficient to rescue social memory formation in Df(16)A mice. We postulate that this rescue at least partially relied on the restoration of CB1R-plasticity by permitting action potential firing in CA2 PNs. Indeed, in a similar animal model, the acute blockade of TREK channels in slice preparation was sufficient to restore CB1R-mediated plasticity (Loisy et al., 2022).

Altogether, these data show that interneuron density is reduced in both a pharmacological and a genetic model of schizophrenia. These studies also revealed that intrinsic properties of both INs and PNs can be altered, thus resulting in specific alterations in CA2 PN activity. Schizophrenia englobes multiple pathologies and using different models might be necessary in order to reveal the different facets of this complex set of diseases.

## 4. Conclusion

Despite an important lag in the number of studies investigating CA2 area in comparison to the other hippocampal regions, significant progress has been made during the last decade in understanding the role of CA2 in controlling the activity and function of the hippocampus in normal conditions. Recent studies have also revealed how alterations in CA2 neurons during diverse pathological states can result in aberrant hippocampal activity and social memory deficits. Gain-of-function (assessed as an increased CA2 pyramidal cells firing such as in AD, ASD, and epilepsy) and lost-of-function (MS, Schizophrenia) have been reported and they both ultimately result in social cognition deficits. This suggests that a fine, bidirectional tuning of CA2 activity is required for the normal function of this region. Unquestionably, inhibition plays a major role in controlling CA2 pyramidal cell activity in normal condition, and alterations in inhibitory transmission and plasticity likely contributes to the deficits in hippocampal function observed during these neurodegenerative and neurodevelopmental disorders. Alterations have often been reported in PV+ and CCK+ interneurons, but other classes of interneurons are also affected. Thus, understanding the precise contribution of diverse populations of interneurons during each specific pathology is one of the challenges of future studies.

## Author contributions

Both authors listed have made a substantial, direct, and intellectual contribution to the work, and approved it for publication.
